# Fingerprinting the Asterid Species Using Subtracted Diversity Array Reveals Novel Species-Specific Sequences

**DOI:** 10.1371/journal.pone.0034873

**Published:** 2012-04-09

**Authors:** Nitin Mantri, Alexandra Olarte, Chun Guang Li, Charlie Xue, Edwin C. K. Pang

**Affiliations:** 1 School of Applied Sciences, Health Innovations Research Institute, RMIT University, Melbourne, Victoria, Australia; 2 Division of Chinese Medicine, School of Health Sciences, Health Innovations Research Institute, RMIT University, Melbourne, Victoria, Australia; James Cook University, Australia

## Abstract

**Background:**

Asterids is one of the major plant clades comprising of many commercially important medicinal species. One of the major concerns in medicinal plant industry is adulteration/contamination resulting from misidentification of herbal plants. This study reports the construction and validation of a microarray capable of fingerprinting medicinally important species from the Asterids clade.

**Methodology/Principal Findings:**

Pooled genomic DNA of 104 non-asterid angiosperm and non-angiosperm species was subtracted from pooled genomic DNA of 67 asterid species. Subsequently, 283 subtracted DNA fragments were used to construct an Asterid-specific array. The validation of Asterid-specific array revealed a high (99.5%) subtraction efficiency. Twenty-five Asterid species (mostly medicinal) representing 20 families and 9 orders within the clade were hybridized onto the array to reveal its level of species discrimination. All these species could be successfully differentiated using their hybridization patterns. A number of species-specific probes were identified for commercially important species like tea, coffee, dandelion, yarrow, motherwort, Japanese honeysuckle, valerian, wild celery, and yerba mate. Thirty-seven polymorphic probes were characterized by sequencing. A large number of probes were novel species-specific probes whilst some of them were from chloroplast region including genes like *atpB*, *rpoB*, and *ndh* that have extensively been used for fingerprinting and phylogenetic analysis of plants.

**Conclusions/Significance:**

Subtracted Diversity Array technique is highly efficient in fingerprinting species with little or no genomic information. The Asterid-specific array could fingerprint all 25 species assessed including three species that were not used in constructing the array. This study validates the use of chloroplast genes for bar-coding (fingerprinting) plant species. In addition, this method allowed detection of several new loci that can be explored to solve existing discrepancies in phylogenetics and fingerprinting of plants.

## Introduction

The Asterids clade of plants is one of the major clades constituting of 1/3 of all known flowering plants. They have been evolutionarily successful and include more than 80,000 species including two of the five most species-rich families of flowering plants [1]. The Angiosperm Phylogeny Group III [2] has grouped asterid species into 97 families and 13 orders based mostly on molecular data from chloroplast genes.

Since early days of civilization, man has used plants as a source of food and medicine. Many of the species used anciently for medicinal purposes belong to the asterid clade of plants. For e.g., sub-fossil remains of *Hyoscyamus niger* L. seeds that dated 5090 BC were found in a filled up Linear Pottery pond in Kueckhoven, Germany [3]. At present, medicinally important species are found in all orders of asterid clade. In fact, using regression analysis to identify the most important families containing medicinal plants, it was found that three asterid families *viz.*, Asteraceae (1), Apiaceae (2), and Lamiaceae (9) ranked among the top medicinal plant families in North America [4]. Asteraceae (1) and Lamiaceae (5) also ranked high in an analysis of Mexican pharmacopoeia [5].

Some popular medicinal species from asterid clade grouped into orders include Cornales (cornus), Ericales (blueberries, tea), Garryales (Eucommia, silktassels), Gentianales (snakeroot, gentians, star jasmine), Solanales (ashwagandha, belladonna, goji berry), Lamiales (red sage, motherwort, tulsi), Aquifoliales (hollies, yerba mate), Apiales (holy ghost, rice-paper plant, Ligusticum), Dipsacales (honeysuckle, valerian), and Asterales (wormwood, codonopsis, Chinese bellflower). One of the basic requirements for successful use of these plants for medicinal purposes is accurate identification of the species. Traditionally, these species were identified on the basis of morphological features. However, as it is difficult to distinguish between certain species purely based on morphology [6], chemical and molecular identification techniques were developed to complement morphological identification. A limitation of chemical analysis techniques is that the chemical composition of these plants varies with environmental effects such as harvest seasons, plant origins and drying procedures [7], [8]. Species identification using genetic diversity is more reliable as genomic information is more specific and does not readily change with environmental factors.

Species identification using genetic diversity has involved techniques such as Amplified Fragment Length Polymorphism (AFLPs), Randomly Amplified Polymorphic DNA (RAPDs) and Restriction Fragment Length Polymorphism (RFLPs) [9], [10]. These techniques utilize the polymerase chain reaction (PCR) for their basis of operation. PCR based techniques are highly sensitive, cheap, accurate and are unaffected by environmental factors. The results are however affected by factors such as the PCR temperature conditions, primers and buffers used [11]. Moreover, as PCR based techniques rely on gel electrophoresis that is a time consuming and labor intensive process, they are not feasible for large scale fingerprinting operations [12], [13]. The resolution of electrophoresis is inadequate for larger DNA fragments, and separation efficiency is poor for small fragments [14].

The development of microarray technique has facilitated plant identification by allowing large scale genotyping/fingerprinting of plants. Diversity array technology (DArT™) developed in 2001 [13] uses genome complexity reduction and microarray technology to identify DNA polymorphisms between species. DArT™ can be used to examine the presence of specific sequences in target DNA samples without prior knowledge of sequence information [13], [15]. However, the genome complexity reduction technique used in DArT™ is insufficient and as a result output data would contain many irrelevant features [16].

As advancement over DArT™, we developed a novel subtractive suppression hybridization (SSH) based microarray technique called the subtracted diversity array (SDA) in 2007 [17]. Compared with diversity DArT™, the SDA technique resulted in a substantial enrichment for polymorphic sequences as a result of the elimination of highly conserved genomic DNA (gDNA) sequences through SSH. The prototype SDA we developed by subtracting gDNA of five non-angiosperm species from gDNA of 49 angiosperm species (including 46 medicinal herbs) was capable of fingerprinting plants up to family level for the species used to make the SDA [18]. Moreover, it could also fingerprint plants that were not used to make the SDA up to the clade level.

The prototype SDA was successful but its discriminatory power was not sufficient to fingerprint all medicinal plants belonging to angiosperms. Two strategies were suggested to increase the discriminatory power of SDA [18]. One was to develop a larger SDA from more angiosperm and non-angiosperm species and other was to develop clade-specific SDA for each of the angiosperm clades. Considering there are over 80,000 species in clades such as Asterids and Rosids, the latter option seems more feasible. This study reports the development, validation and use of Asterid-specific SDA that is capable of fingerprinting medicinally important asterid species. Further, we sequenced selected spots that were discriminatory for the species tested to reveal their identity.

## Results and Discussion

### Validation of Asterids-specific SDA

The Asterids-specific SDA was first validated to determine the efficiency of the Asterids Clade-specific gDNA subtraction. For this, the gDNA pool of 67 species representing the Asterid clade (AC) and gDNA pool of 104 species representing the non-asterid angiosperms and non-angiosperms (NA) were separately hybridized onto the Asterid-specific SDA. Only 1 out of 283 subtracted fragments (probes on the array) hybridized with the NA gDNA pool indicating a nearly perfect subtraction. This subtraction efficiency was much better than the 97% efficiency obtained during preparation of the original SDA [17]. Further, 33 out of 283 spots did not hybridize with the AC gDNA pool from which they were prepared. This may be due to ‘dilution effect’ (low frequency sequences remain undetected in complex targets) as described [17]. Alternatively, these 33 subtracted fragments may be of bad quality, thus affecting hybridization. The stringent hybridization and analysis conditions used may have probably eliminated these bad fragments.

The specificity of the Asterids-specific SDA was also validated by hybridizing the gDNA of five species representing the non-Asterids clades in the plant kingdom. The species tested include *Magnolia denudata* (Magnoliaceae, Magnoliids); *Coix lacryma-jobi* (Poaceae, Monocots); *Ranunculus ternatus* (Ranunculaceae, Eudicots); *Agrimonia pilosa* (Rosaceae, Rosids); and *Sphagnum australe* (Sphagnaceae, Non-angiosperms). The gDNA of these species also hybridised to only 1 out of 283 spots as observed with using the gDNA pool of all non-asterid and non-angiosperm species (driver pool). This further supports the claim that the SDA constructed is specific for Asterid species.

### Capacity of Asterids-specific SDA to fingerprint various Asterids species

Twenty-five Asterids species representing 20 families and 9 orders within the clade were hybridized onto the array to reveal the level of species discrimination (Table 1). The microarray experiments were carried out according to MIAME guidelines and all data has been deposited in Gene Expression Omnibus (GSE31242). All the 25 asterid species tested using this array generated different hybridization patterns allowing discrimination. *Cornus spp.* (family Cornaceae) hybridized to least number of probes (1/283) whilst *Coffea arabica* (family Rubiaceae) hybridized to most number of probes (80/283). Cornus was expected to hybridize to less number of probes as it belongs to order Cornales which is an out-group or sister to all other orders of the Asterid clade [19]. The hybridization of *Coffea arabica* to large number of probes may be attributed to its huge genome size (1300 Mb) and allotetraploid nature [20].

**Table 1:** List of 25 Asterid species hybridized onto the Asterid-specific SDA to reveal its level of species discrimination.

Species	Family	Order
*Cornus spp*	Cornaceae	Cornales
*Impatiens mix*	Balsaminaceae	Ericales
*Camellia sinensis*	Theaceae	Ericales
*Coffea arabica*	Rubiaceae	Gentianales
*Gardenia jasminoides*	Rubiaceae	Gentianales
*Tracheospermum jasminoides*	Apocynaceae	Gentianales
*Valeriana officinalis*	Valerianaceae	Dipsacales
*Sambuscus nigra*	Adoxaceae	Dipsacales
*Lonicera japonica*	Caprifoliaceae	Dipsacales
*Rehmannia glutinosa*	Phrymaceae	Lamiales
*Scrophularia nodosa*	Scrophulariaceae	Lamiales
*Digitalis purpurea*	Plantaginaceae	Lamiales
*Forsythia suspensa*	Oleaceae	Lamiales
*Vitex agnus-castus*	Lamiaceae	Lamiales
*Leonurus cardiaca*	Lamiaceae	Lamiales
*Symptum spp*	Boraginaceae	Lamiales
*Codonopsis* spp	Campanulaceae	Asterales
*Platycodon grandiflorus*	Campanulaceae	Asterales
*Taraxacum officinale*	Asteraceae	Asterales
*Achillea millefolium*	Asteraceae	Asterales
*Angelica archangelica*	Apiaceae	Apiales
*Tetrapanax papyriferus*	Araliaceae	Apiales
*Ilex paraguariensis*	Aquifoliaceae	Aquifoliales
*Lycium barbarum*	Solanaceae	Solanales
*Withania somnifera*	Solanaceae	Solanales

Interestingly, three species (*Cornus spp.*; *Gardenia jasminoides*; *Lonicera japonica*) that were not used to make initial gDNA pool for subtraction also hybridized to probes on the array and produced unique fingerprints allowing their differentiation. This result corroborates the report by [18] that the SDA can be used to fingerprint species from the ‘tester’ group that were not used in initial gDNA subtraction. This ability of the SDA is advantageous over other subtraction suppressive hybridization (SSH) based arrays that employ pair-wise subtraction and can only discriminate the species used to construct the array [16]. Moreover, the broad subtraction approach followed in SDA is efficient, economical, and less labor intensive than other DNA based fingerprinting methods [21].

Further, 142 probes out of 283 (50%) hybridized to the restriction digested gDNA of the 25 Asterids species tested and revealed polymorphism between them. This polymorphism rate is similar to the 42.4% reported for other SSH-based arrays [16] but considerably higher than 3–27% reported for Diversity Array Technology (DArT™) [13], [22], [23], [24]. The possible reason is that in the SSH based arrays and SDA, common sequences are eliminated by the subtraction process, thus enriching the probe library with polymorphic sequences for the species being investigated.

### Statistical analysis

**Figure 1:**
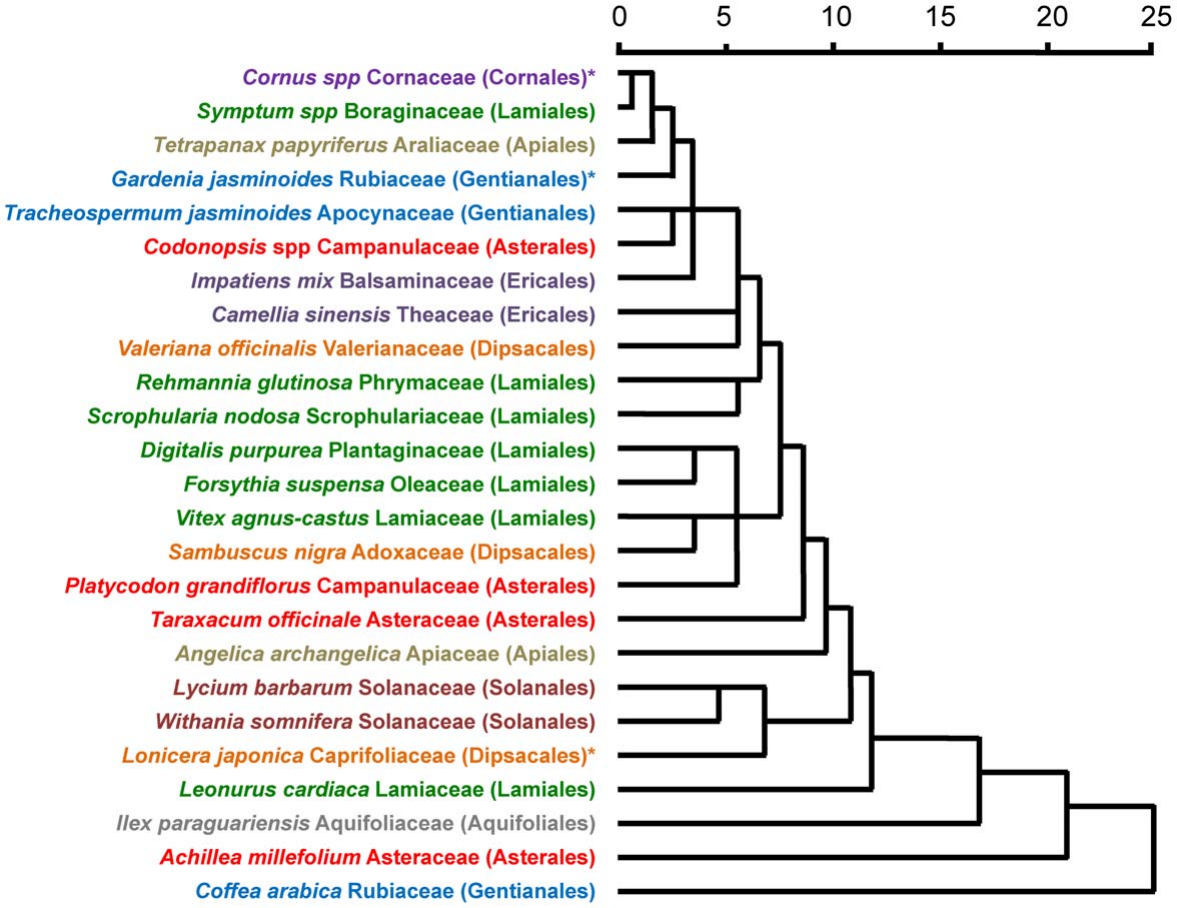
Hierarchical clustering of the 25 Asterid species using the hybridization signal from probes on the Asterid-specific array. Clustering was performed using average median Log_2_ values of good features and ‘between groups linkage’ and ‘Euclidean distance’. The botanical name of each species in ‘italics’ is followed by its family in ‘normal’ font and order in the ‘brackets’. Species belonging to same order have been highlighted with same font color. Species marked with asterisk (*) were not used in the construction of the Asterid-specific array.

The relationship between the 25 Asterids species was examined by constructing a dissimilarity dendrogram using the median Log_2_ values of probes that passed the quality control measures. The hierarchical cluster analysis with Euclidean distance and between-groups linkage is presented in Figure 1. This figure demonstrates that the Asterid-specific SDA has the ability to discriminate Asterid species belonging to same and different families and orders within the Asterids clade. It also shows that the array generally groups closely related species together. However, some species from the same order did not group together (example Gentianales, Ericales, Asterales). Further, whilst some species of same families grouped together (example, *Lycium barbarum* and *Withania somnifera*; *Platycodon grandiflorus* and *Codonopsis spp*), others did not (example, *Gardenia jasminoides* and *Coffea arabica*; *Taraxacum officinale* and *Achillea millefolium*). The position of various families and orders in the dendrogram was also not same as the generally accepted model [2]. The possible reason is because the probes on Asterids array were generated by subtraction of restriction digested gDNA pools. The species used for pooling were chosen from those that were available to us. Species for some families or orders were either missing or under-represented compared to species from other families and orders (Table S1). Moreover, we did not use representative or type species for each of the families or orders because a lot of type species were not medicinally important. Further, the sequence of the probes on Asterid array was not known and the 283 clones selected for fingerprinting were randomly picked merely based on size variation. It should be noted that the primary objective of the current study was to develop a microarray to fingerprint the medicinal species belonging to the Asterids clade and not to explore the phylogenetic relationship of the Asterids species.

A Principal Component Analysis (PCA) was performed using SPSS version 17.0 to identify the probes that caused the majority of variance in the data. The analysis extracted six factors with significant Eigenvalues but the first two factors explained the majority (56.45%) of the variance in the data. The probes that explained the maximum variance hybridized to the DNA from most of the 25 Asterid species tested. The variance was explained by the difference in signal intensities (median Log_2_ values) for the particular probe.

### Sequence characterization of selected probes

The hybridization patterns of all the 25 asterid species tested were compared to identify important distinguishing spots/probes. The probes were identified based on two criteria: a) probes that showed most variance between the species assessed (based on PCA analysis), and b) probes that specifically hybridized to a particular species or species from the same family and order. The list of these probes along with their significance is presented in Table 2. As seen in the table, a number of species-specific probes were identified for commercially important species like *Camellia sinensis* (tea), *Coffea arabica* (coffee), *Taraxacum officinale* (dandelion), *Achillea millefolium* (yarrow), *Leonurus cardiaca* (motherwort), *Lonicera japonica* (Japanese honeysuckle), *Valeriana officinalis* (valerian), *Angelica archangelica* (wild celery), and *Ilex paraguariensis* (yerba mate). These probes can be potentially used as molecular markers to identify these species. The identification of a large number of species-specific probes reconfirms the importance of the SDA technique for fingerprinting plants with little or no genomic information.

**Table 2:** Important probes selected after comparing hybridization patterns of the 25 Asterid species assessed.

Clone ID	Importance
***All Asterids***	
5TP230, 5TP235, 5TP111, 5TP286, 5TP296, 5TP218, 4TP117, 5TP249, 5TP179, 5TP236	Probes from PCA analysis revealing maximum amount of variation. These probes hybridized with most of the Asterid species assessed.
***Order: Ericales***	
5TP204, 5TP228	Specifically hybridized to *Camellia sinensis* (Theaceae)
5TP104	Hybridized only to *Camellia sinensis* (tea) and *Coffea arabica* (coffee). Higher signal strength in coffee than tea.
***Order: Gentianales***	
4TP143, 4TP168, 5TP115, 5TP250, 5TP282, 4TP122, 5TP112, 5TP113, 5TP114, 5TP248	Specifically hybridized to *Coffea arabica* (Rubiaceae)
***Order: Asterales***	
4TP138	Specifically hybridized to *Taraxacum officinale* (Asteraceae)
4TP174, 5TP162, 5TP212, 5TP281, 5TP292, 4TP162, 4TP220, 5TP247, 5TP245, 5TP147, 5TP161, 5TP257, 5TP291	Specifically hybridized to *Achillea millefolium* (Asteraceae)
4TP131, 4TP155, 5TP274, 4TP153, 4TP187	Specifically hybridized to *Leonurus cardiaca* (Lamiaceae)
***Order: Solanales***	
4TP140, 4TP132, 4TP103	Hybridized with both Solanaceae species (*Withania somnifera* & *Lycium barbarum*). Did not hybridize with any other species tested except *Lonicera japonica* (Caprifoliaceae)
***Order: Dipsacales***	
4TP170, 5TP110, 5TP275	Specifically hybridized to *Lonicera japonica* (Caprifoliaceae)
4TP173	Specifically hybridized to *Valeriana officinalis* (Valerianaceae)
5TP106	Hybridized only to *Lonicera japonica* (Caprifoliaceae) and *Sambuscus nigra* (Adoxaceae)
***Order: Apiales***	
4TP163, 5TP135	Specifically hybridized to *Angelica archangelica* (Apiaceae)
***Order: Aquifoliales***	
4TP106, 4TP111, 4TP178, 5TP219, 5TP227, 4TP109, 4TP158, 5TP215, 5TP226, 5TP238, 5TP239, 5TP240	Specifically hybridized to *Ilex paraguariensis* (Aquifoliaceae)

From the important probes listed in Table 2, 37 probes representing all categories were selected for sequencing. The sequences were edited using Bioedit software and characterized using Genome Sequence Survey, EST_others and Chromosome databases in NCBI BLAST. Interestingly, 14 probe sequences did not have any match in the NCBI database suggesting these are novel sequences. More importantly, these 14 probes hybridized only to a single species suggesting these are novel species-specific sequences. Four probes were specific for *Achellia millefolium* (HE565561, HE565564, HE565578, HE565592), three were specific for *Coffea arabica* (HE565559, HE565572, HE565576), two specific for *Camellia sinensis* (HE565563, HE565567) and one each specific for *Ilex paraguariensis* (HE565579, HE565580), *Leonurus cardiaca* (HE565587), *Angelica archangelica* (HE565588), and *Valeriana officinalis* (HE565591).

#### Sequence characterization of PCA spots

The identity match of remaining 23 probes (out of the 37 sequenced) is presented in Table S2. These results are also interesting. The probes that hybridized to most of the Asterid species tested (from PCA analysis) were mostly identical to different chloroplast genes that have been commonly used for fingerprinting plants [19]. Importantly these probes were identical to chloroplast genes of various Asterid species. Two probes were ≥95% identical to RNA polymerase β-chain (*rpoB*), one was 84–93% identical to *rpoB*, one was 92% identical to ATPase β subunit (*atpB*), and one was ≥90% identical to NADH plastoquinone oxidoreductase or NADH dehydrogenase (*ndh*) of different Asterid species. Although the *atpB* and *ndh* genes have been frequently used for fingerprinting and phylogenetic analysis of various plant species including Asterid species [25], [26], the *rpoB* has only been recently used for fingerprinting some microbial species [27], [28]. The success of using the *rpoB* gene for fingerprinting microbes previously and Asterid species in this study could mean that this gene can be explored to solve some of the existing discrepancies in the phylogenetics of Asterid species [2]. It is also important to note that we did not deliberately choose these chloroplast genes for fingerprinting. The subtraction process we used to subtract common sequences between Asterid and all other non-asterid species actually selected these chloroplast genes that were specific for asterid species. This again highlights the importance of SDA technique for fingerprinting.

Further, probe 5TP230 (HE565568) that hybridized to most of the Asterid species tested (from PCA analysis) was 95–99% identical to chloroplast of many asterid species. Interestingly, on the EST database, 5TP230 was 96% identical to *N. benthamiana* glycosyltransferase enzyme. According to our knowledge, gycosyltransferase has never been used to fingerprint plant species and only in one recent study it was identified as a marker to differentiate *Bacillus anthracis* from other members of the *B. cereus* group [29]. Remarkably enough, they discovered a glycosyltransferase clone to be *B. anthracis*-specific marker when they used Suppression subtractive hybridization (SSH) to subtract *B. cereus* DNA from *B. anthracis* DNA.

Additionally, the sequence of probe 5TP179 (HE565562) that hybridized to most of the Asterid species tested (from PCA analysis) was 100% identical to only one *Coffea arabica* sequence from the GSS database. This probe sequence was also 37% identical to *C. canephora* pericarp in the EST database. To our knowledge, pericarp sequences have never been used to fingerprint plants. Also, the sequence of probe 5TP218 (HE565565) that hybridized to most of the Asterid species tested (from PCA analysis) had no significant match in the NCBI database. Only 22% of the sequence was 84% identical to *Brachypodium* (monocot) sequence from the GSS. Since both these probes hybridized to most of the asterid species tested with different signal intensities, the genomic region corresponding to these probes should be further explored for fingerprinting plants.

#### Sequence characterization of species/family/order-specific spots

From Table S2, the sequence identity of the probes that were species/family/order-specific and showed at least some sequence similarity in the NCBI database is discussed here. Out of the three probes that were specific for *Lonicera japonica*, 87% sequence of 5TP110 (HE565558) was 96% identical to ATPase β subunit (*atpB*) gene of many Lamiales species, and ≥98% sequence of 4TP170 (HE565590) was identical to NADH plastoquinone oxidoreductase subunit 2 (*ndhB*) of many plant species. As explained above, *atpB* and *ndh* are chloroplast genes that have been extensively used for fingerprinting and phylogenetic analysis of plants. Importantly, the third *Lonicera japonica*-specific probe (5TP275 - HE565574) had no significant match in the database. The highest match was 59% of the sequence was 82% similar to *Poncirus trifoliata* citrus tristeza virus resistance gene. Therefore this probe is potentially a novel marker for *Lonicera japonica*.

Among the probes that were specific for *Ilex paraguariensis*, 98% sequence of 5TP219 (HE565566) was 85–88% similar to only 2 species, namely, *Lactuca sativa* and *Barnadesia spinosa*. Interestingly, both these species belong to family Asteraceae whilst *Ilex paraguariensis* is a member of family Aquifoliaceae. The sequence of other probe specific for *Ilex paraguariensis*, 4TP106 (HE565579), did not have any significant match in the database. Only 10% of the sequence was 92% identical to a *Vitis vinifera* (grape vine) shotgun sequence. Both these probes are therefore novel markers for *Lonicera japonica*.

The probes 4TP131 (HE565582) and 5TP274 (HE565573) were found to be specific for *Leonurus cardiaca*. The sequence of 4TP131 had no significant match in the database. However, 97% of the sequence was 71% similar to *Glycine max* (soybean). Also, 73–76% sequence was 70–72% identical to copia-type pol polyprotein in soybean and *Beta vulgaris* (beet). Interestingly, 98% sequence of 5TP274 as well was 77% identical to soybean and a bit lower matches to other legumes, namely, lotus, chickpea, and medicago. The legumes however belong to Fabaceae family of Rosids clade whilst *Leonurus cardiaca* belongs to Lamiaceae family of Asterids. The similarity of *Leonurus cardiaca* with these species needs further investigation. However, it should be noted that there is abundance of sequence information for these legumes as they are either commercially important (chickpea, soybean) or model species (lotus, medicago). Comparatively, there is little sequence information available for the Lamiaceae species which may have resulted in no match being found. Nevertheless, both these probes can be used as specific markers for identification of *Leonurus cardiaca*.

No significant match was found in the database for sequence of the probe 5TP135 (HE565560) that was specific for *Angelica archangelica*. Only 27% of the sequence was 91% identical to ATPase β subunit (*atpB*) gene of some Lamiales species. Similarly, no significant match was found in the database for the sequence of probe 5TP281 (HE565575) that was specific for *Achellia millefolium*. Only 33–50% sequence was similar to a monocot (maize) and a dicot (soybean) sequence. These probes therefore can potentially serve as markers for *Angelica archangelica* and *Achellia millefolium*.

The probe 4TP138 (HE565584) was found to be specific for *Taraxacum officinale* (dandelion) of family Asteraceae. The sequence of 4TP138 was ≤92% identical to chloroplast of lettuce and other Asteraceae species. Another important observation was that probes 4TP132 (HE565583) and 4TP140 (HE565585) hybridized specifically to both the Solanaceae species tested (*Withania somnifera* and *Lycium barbarum*) and *Lonicera japonica* that belongs to Caprifoliaceae family of order Dipsacales. Interestingly, the sequence of both these probes is 95–97% identical to chloroplast of various Solanaceae species. Further study is needed to determine the relationship between *Lonicera japonica* and species from family Solanaceae.

Finally, we present key results for two prominent commercial species: tea (*Camellia sinesis*) and coffee (*Coffea arabica*). The probe 5TP104 (HE565556) hybridized specifically to tea and coffee from the 25 Asterid species tested. The sequence of this probe was found to be 98% identical to only one *C. arabica* sequence in the GSS database. Since both these plants are caffeine producing, further investigation is needed to determine the importance of this sequence in these plants. Also, probes 4TP168 (HE565589) and 4TP143 (HE565586) were specific for *C. arabica.* Interestingly, 70–80% sequence of probe 4TP143 was found to be 87–94% identical to only five *C. arabica* sequences including one ISSR marker. Moreover, 4TP168 was 96% identical to only one *C. arabica* sequence in the NCBI database. The identification of a *C. arabica*-specific ISSR marker yet again highlights the significance of the SDA method for isolating species-specific sequences for fingerprinting. Importantly, it identifies novel species-specific markers like 4TP168 that only matched to one sequence in the NCBI database.

### Conclusions

Accurate identification of herbal plant samples is crucial in quality control of herbal medicine. In this study, we have successfully used SDA technique to develop an Asterids-specific microarray that could fingerprint 25 Asterid species (mostly medicinal plants) representing 20 families and 9 orders within the clade. An important feature of this microarray was that it could fingerprint three Asterid species that were not used to construct it. A number of species-specific probes were identified for commercially important species like tea, coffee, dandelion, yarrow, motherwort, Japanese honeysuckle, valerian, wild celery, and yerba mate. Sequencing of these important probes revealed that a large number of probes were novel species-specific probes whilst some of them were from chloroplast region including genes like *atpB*, *rpoB*, and *ndh* that have been extensively used for fingerprinting and phylogenetic analysis of plants. In addition, we have identified other genes like glycosyltransferase and copia-type pol polyprotein, and a sequence related to pericarp that can be explored for fingerprinting plants in the future. The results reconfirm the significance of the SDA technique in fingerprinting wide range of plants.

## Materials and Methods

### Plant material and genomic DNA extraction

Leaf tissues of 67 species representing the Asterid clade (AC) and 104 species representing the non-asterid angiosperms and non-angiosperms (NA) were obtained from the herbarium at Southern Cross University Plant Science, NSW, Australia. High quality genomic DNA (gDNA) was extracted from these specimens using DNeasy® Plant Mini Kit (Qiagen Inc., Australia). The quantity of DNA was measured using Eppendorf spectrophotometer whilst the quality/integrity was assessed by 1.5% agarose gel electrophoresis.

### Genomic DNA subtraction and library construction

The gDNA was pooled into two separate groups, AC and NA, by mixing equal quantities of DNA from individual species belonging to that group. Subsequently, 4 µg of pooled gDNA from each group was restriction digested in a 50 µL reaction using 5U of *Hae*III and *Alu*I (New England Biolabs). As previously described (Jayasinghe *et al*., 2007), the digested NA gDNA pool was subtracted from digested AC gDNA pool to isolate AC-specific DNA using the Clontech PCR-Select™ cDNA Subtraction Kit (Clontech, Mountain View, CA). The AC-specific DNA fragments were cloned into pGEM-T® Easy Vector (Promega, Madison, WI) and transformed into *Escherichia coli* JM109 competent cells (Promega, Madison, WI), resulting into 283 clones with insert of 250–750 bp.

### AC-specific clone amplification and SDA printing

The 283 AC-specific DNA clones were amplified in 100 µL PCR reactions using Clontech nested primers as described (Jayasinghe *et al*., 2007). PCR products were transferred into V-bottom polypropylene 96-well plates and purified by ethanol/sodium acetate precipitation before resuspending in 50% dimethyl sulfoxide (DMSO). The control spots on the SDA included printing control (Cy-3) and negative controls *viz*., printing buffer (50% DMSO), nested primer 1 and 2R (Clontech, Mountain View, CA), and pGEM-T® Easy Vector (Promega, Madison, WI) digested with *Hae*III and *Alu*I. The 283 AC-specific DNA fragments along with controls were spotted on a Corning GAPS II coated slides (Corning Incorporated Life Sciences, Acton, MA) using BioRobotics MicroGrid II Compact (Genomics Solutions, Ann Arbor, MI) microarray spotter at RMIT University, Australia.

### Target synthesis

The SDA was first validated by testing for the success of NA gDNA subtraction by separately hybridizing DNA fragments from pooled AC and pooled NA onto the array. Secondly, the array was tested for the ability to differentiate a population of 25 species representing 20 families and 9 orders within asterid clade (all 25 medicinal herbs). The preparation of targets in all cases involved the double digestion of 0.5 µg of pooled total DNA with *Alu*I and *Hae*III, and purification using Qiaquick® PCR Purification Kit (Qiagen Inc.). Biotin-11-dUTP was then incorporated into restriction digested gDNA fragments using the Biotin DecaLabel™ DNA Labeling Kit (Fermentas, ON, Canada) following the manufacturer's guidelines. However, the incubation time was increased to 20 h, the reaction stopped with 1 µl 0.5 M EDTA, pH 8.0 and labelled gDNA fragments were purified using Qiaquick® PCR Purification Kit.

### Hybridization of the SDA

The SDA slides were pre-hybridized for 45 min at 42°C in a pre-warmed solution containing 5× standard saline citrate (SSC), 0.1% sodium dodecyl sulphate (SDS), 1% bovine serum albumin (BSA) and 25% formamide. The slides were rinsed twice with sterile MilliQ water and immediately dried with an air gun.

The biotin-labelled targets (dried to 16 µL) were added to 17.5 µL of fresh 2× Hybridization buffer (250 µL of formamide, 250 µL of 10× SSC, 10 µL of 10% SDS), 0.5 µL of 5 µg/µL Human Cot1 DNA (Sigma-Aldrich, St Louis, MO), 0.5 µL of 10 mg/mL Poly A (Sigma-Aldrich) and 0.5 µL of 10 mg/mL salmon sperm DNA (Sigma-Aldrich). The mixture was denatured at 100°C for 2 min and immediately applied onto the array under a 22×25-mm lifter slip (Grale Scientific, Victoria, Australia). The slides were then placed in waterproof, humidified hybridization chambers (Corning Incorporated Life Sciences) and incubated overnight in a 42°C water bath. Following hybridization, the slides were washed twice for 5 min in 500 mL Wash buffer 1 (1× SSC with 0.1% SDS), once for 5 min in 500 mL Wash buffer 3 (0.1× SSC with 0.1% SDS), and once for 5 min in 500 mL Wash buffer 4 (0.1× SSC). Subsequently the slides were transferred to 500 mL of 6× SSPE-T buffer (0.9 M NaCl, 0.06 M NaH_2_PO_4_.H_2_O, 0.006 M EDTA, 0.005% Triton X-100, pH 7.4) without allowing them to dry.

The biotinylated DNA targets bound on the array were then labelled with fluorescent FluoroLink™ streptavidin-labelled Cy3 dye (Amersham Pharmacia, UK) using a biotin–streptavidin system. Briefly, 200 µL of a Detection solution (0.5 µL of 0.8 µg/µL streptavidin-labelled Cy3, 0.8 µl of 25 µg/µL BSA, made to 200 µL with 6× SSPE-T) was applied directly onto the array surface and a 22×25-mm lifter slip was placed over it to evenly distribute the solution on the array. The slides were placed in hybridization chambers, wrapped in aluminum foil and incubated at 37°C for 1 h in the dark. Finally, the slides were washed thrice in 6× SSPE-T for 5 min and rinsed with sterile MilliQ water before being dried with an air gun. All hybridizations were performed with six technical replicates (corresponding to six sub-arrays) and two biological replicates, resulting in 12 data points for each array feature.

### Scanning and Data Analysis

Slides were scanned with a ScanArray Gx (PerkinElmer Life and Analytical Sciences, Downers Grove, IL) microarray scanner in conjunction with the supplied software. The slides were scanned with a resolution of 5 µm at 532 nm (Cy3, green laser) and at 55% photomultiplicator (PMT) gain whilst keeping background noise low. The scanned array was quantified using PerkinElmer ScanArray Express software v 2.0. The program individually quantified the signal intensity at each probe and normalized the data using the adaptive circle and LOWESS functions. Probes which did not hybridize were automatically flagged by the scanning software and labelled as ‘bad’. Manual flagging was used to remove spots displaying inconsistent hybridization such as ‘donut’ spots. ‘Good’ probes were accepted as having a mean ‘signal to noise ratio’ (SNR) value of greater than 5 in more than half of the technical replications.

Data analysis included subtracting the background from median signal intensity for each feature, log_2_ transformation and combining technical replicates by taking average. Subsequently, the signal intensities and flag values of the two biological replicates were compared and average signal intensities were calculated for only those features that were flagged ‘Good’ in both the replicates. The values of features that had a ‘Bad’ flag in either or both the replicates were converted to zero. Finally, SPSS version 17.0 (SPSS Inc., Chicago, IL) was used to examine relationships between the 25 asterid species by constructing a dissimilarity dendrogram using hierarchical cluster analysis with Euclidean distance and between-groups linkage. A Principal Component Analysis (PCA) was also performed using SPSS version 17.0 to identify the probes that reveal maximum difference between the species assessed.

### Sequence characterization of selected features

Thirty-seven probes were selected for sequencing based on PCA analysis and specificity of the probes to particular species or families. The probes were amplified in a 50 µL reaction with 2U *Taq* DNA polymerase (Invitrogen, Carlsbad), 1.5 mM MgCl_2_, 200 µM dNTPs, 0.4 µM of Nested Primers 1 and 2 (Clontech). The cycling conditions were one cycle of 94°C for 3 min, 35 cycles of 94°C for 30 s, 60°C for 45 s and 72°C for 45 s, and a final extension of 72°C for 5 min. The PCR products were purified using Qiaquick® PCR Purification Kit and sequenced by Macrogen Inc. (Seoul, Korea). The sequences were analyzed using Genome Sequence Survey, EST_others and Chromosome databases in NCBI BLAST (www.ncbi.nlm.nih.gov/BLAST/). All sequences have been deposited in EMBL Nucleotide Sequence Database (Accession number HE565556 to HE565592) (Table S3).

## Supporting Information

Table S1 **List of species used as driver and tester in suppression subtractive hybridisation to get asterid-specific sequences.** Leaf tissues of 67 species representing the Asterid clade (AC) and 104 species representing the non-asterid angiosperms and non-angiosperms (NA) were used to construct the tester and driver pools, respectively.
(XLS)

Table S2 **Sequence characterization of probes selected after comparing hybridization patterns of the 25 Asterid species assessed.** Thirty-seven probes were selected for sequencing based on PCA analysis and specificity of the probes to particular species or families. The sequences were analyzed using Genome Sequence Survey, EST_others and Chromosome databases in NCBI BLAST.
(DOCX)

Table S3 **EMBL Accession numbers of subtracted clones that were sequenced.** Thirty-seven probes were selected for sequencing based on PCA analysis and specificity of the probes to particular species or families.
(XLSX)
