# Supplementary material for: Fingerprinting the Asterid Species Using Subtracted Diversity Array Reveals Novel Species-Specific Sequences
Source: PLoS One. 2012 Apr 9;7(4):e34873. doi: 10.1371/journal.pone.0034873 (PMC3322160; doi:10.1371/journal.pone.0034873)
Supplement: Table S2 — Sequence characterization of probes selected after comparing hybridization patterns of the 25 Asterid species assessed. Thirty-seven probes were selected for sequencing based on PCA analysis and specificity of the probes to particular species or families. The sequences were analyzed using Genome Sequence Survey, EST_others and Chromosome databases in NCBI BLAST. (DOCX) [file pone.0034873.s002.docx]

Table S2. Sequence characterization of probes selected after comparing hybridization patterns of the 25 Asterid species assessed

| **Clone ID** | **EMBL Accession number** | **Significance** | **Sequencing Results**  **(% match with GenBank^®^ ID)** |
| --- | --- | --- | --- |
| **4TP117** | HE565581 | hybridized to most species from PCA | 91-92% identical to chloroplast (NADH plastoquinone oxidoreductase) of *Antirrhinum majus* (GQ996981.1)*, Olea europaea* (GU228899.1). <90% identical to chloroplast of many other asterid species (GQ997645.1; GQ997313.1; AF384484.2; EF044213.1; EU385311.1; EU385285.1) |
| **5TP179** | HE565562 | hybridized to most species from PCA | 100% identical to *Coffea arabica* GSS (ED795417.1). 37% identical to *C. canephora* pericarp (DV679008.1) |
| **5TP218** | HE565565 | hybridized to most species from PCA | No significant match found. 22% sequence is 84% identical to Brachypodium (monocot) (GS137090.1) |
| **5TP230** | HE565568 | hybridized to most species from PCA | 100% sequence is 95-99% identical to chloroplast of many asterid species (GU120098.1; EU549769.1; DQ383815.1; EZ182968.1; AY582139.1; AM087200.3; AB240139.1; AB237912.1; AJ316582.1; DQ347959.1; GU228899.1). 96% identical to *Nicotiana benthamiana* glycosyltransferase enzyme (EH369124.1) |
| **5TP235** | HE565569 | hybridized to most species from PCA | 92% identical to *ATPase* *B* subunit of lot of asterid species (AJ236176.1; AJ236174.1; AJ236173.1; DQ401329.1; AF209551.1; AJ235420.2; AJ236177.1; AJ236175.1) |
| **5TP236** | HE565570 | hybridized to most species from PCA | 84-93% identical to chloroplast (RNA polymerase *B* subunit) of many Asterids (DQ407063.1; AP007232.1; GQ997111.1; DQ407055.1; DQ407056.1; XM_002266815.1; GQ997360.1) |
| **5TP249** | HE565571 | hybridized to most species from PCA | >95% identical to chloroplast (RNA polymerase B-chain) of some asterid species (DQ407063.1; DQ383815.1; EZ349840.1; AP007232.1; EU549769.1; GU120098.1). Upregulated after abiotic stress in some species |
| **5TP286** | HE565577 | hybridized to most species from PCA | >95% identical to chloroplast (RNA polymerase B-chain) of some asterid species (DQ407063.1; EZ349840.1; DQ383816.1; EU549769.1). Upregulated after abiotic stress in some species |
| **4TP170** | HE565590 | Specifically hybridized to *Lonicera japonica* (Caprifoliaceae) | 100% identical to *Antirrhinum majus* NADH plastoquinone oxidoreductase subunit 2 (GQ996979.1). Also >98% identical to chloroplast of many other species (GQ997062.1; GU228899.1; EF207455.1; EF207445.1; EF207444.1; EF207443.1). |
| **5TP110** | HE565558 | Specifically hybridized to *Lonicera japonica* (Caprifoliaceae) | 87% sequence is 96% identical to ATPase B subunit gene of many Lamiales species (DQ401329.1; AJ235420.2; AJ235519.2; AJ236177.1; AJ236176.1). |
| **5TP275** | HE565574 | Specifically hybridized to *Lonicera japonica* (Caprifoliaceae) | No significant match found. 59% is 82% similar to *Poncirus trifoliata* - citrus tristeza virus resistance gene (AF506028.1) |
| **5TP135** | HE565560 | Specifically hybridized to *Angelica archangelica* (Apiaceae) | No significant match found. 27% is 91% identical to ATPase B subunit of some Lamiales (DQ401329.1; AY792770.1; AF209646.1; AF209551.1; AJ235639.2; AJ235625.2) |
| **4TP106** | HE565579 | Specifically hybridized to *Ilex paraguariensis* (Aquifoliaceae) | No significant match found at all |
| **5TP219** | HE565566 | Specifically hybridized to *Ilex paraguariensis* (Aquifoliaceae) | 98% sequence is 85-88% similar to only 2 species - *Lactuca sativa* and *Barnadesia spinosa* - both Asteraceae (DQ383816.1; GE543322.1). *Ilex* is Aquifoliaceae |
| **4TP131** | HE565582 | Specifically hybridized to *Leonurus cardiaca* (Lamiaceae) | No significant match found. However, <71% similar to soybean (AC235394.1) and other legumes (GS877609.1; AP008242.1; FP700158.3). Also, 73-76% sequence is 70-72% identical to copia-type pol polyprotein in soybean (BG510236.1) and *Beta vulgaris* (BI643218.1). |
| **5TP274** | HE565573 | Specifically hybridized to *Leonurus cardiaca* (Lamiaceae) | 98% sequence is 76% identical to *Glycine max* (AC235394.1) and bit lower matches to other legumes – lotus (AP010574.1), chickpea (EU877333.1), medicago (AC160925.39) |
| **5TP281** | HE565575 | Specifically hybridized to *Achillea millefolium* (Asteraceae) | No significant match found. 33-50% is similar to monocot (maize - CC932102.1) and dicot (soybean - AC235460.1) species |
| **4TP138** | HE565584 | Specifically hybridized to *Taraxacum officinale* (Asteraceae) | <92% identical to chloroplast of lettuce and other Asteraceae (DQ383816.1; EZ181346.1; DQ383815.1; GU120098.1). 92% identical to root cDNA clone from *Taraxacum* (GO668374.1). |
| **4TP132** | HE565583 | Specifically hybridized to both Solanaceae species and *Lonicera* *japonica* (Caprifoliaceae) | 95-97% identical to chloroplast (chromosome) couple of Solanaceae species (AJ316582.1; DQ347958.1; BT013543.1; DQ386163.2; AB237912.1). 93-96% identical to ESTs from some Solanaceae (AW649920.1; FS066203.1; AM810929.1) |
| **4TP140** | HE565585 | Specifically hybridized to both Solanaceae species and *Lonicera* *japonica* (Caprifoliaceae) | 97% sequence is 97% identical to chloroplast of Solanaceae species *viz*., *Atropa belladonna* (AJ316582.1), *S. tuberosum* (DQ386163.2), *S. lycopersicum* (AM087200.3), *S. bulbocastanum* (DQ347958.1) and 96% identical to *N. tomentosiformis* (AB240139.1) |
| **5TP104** | HE565556 | Specifically hybridized to *Camellia sinensis* (tea) and *Coffea arabica* (coffee) | 100% sequence is 98% identical to only one *C. arabica* GSS (ED794009.1) |
| **4TP168** | HE565589 | Specifically hybridized to *Coffea arabica* (coffee) | only 1 sequence with 96% identity found in *C. arabica* (ED794293.1) |
| **4TP143** | HE565586 | Specifically hybridized to *Coffea arabica* (coffee) | 70-80% sequence is 87-94% identical to only five *C. arabica* sequences (ED794678.1; ED793750.1; ED794916.1; ED795823.1) including an ISSR marker (AJ318868.1). |
